# Changes in short-term (in-ICU and in-hospital) mortality following intensive care unit admission in adults living with HIV: 2000–2019

**DOI:** 10.1097/QAD.0000000000003683

**Published:** 2023-08-22

**Authors:** Tanmay Kanitkar, Oshani Dissanayake, Nicholas Bakewell, Maggie Symonds, Stephanie Rimmer, Amit Adlakha, Marc C.I. Lipman, Sanjay Bhagani, Caroline A. Sabin, Banwari Agarwal, Robert F. Miller

**Affiliations:** a Intensive Care Unit; b HIV Services, Royal Free Hospital, Royal Free London NHS Foundation Trust; c Centre for Clinical Research, Epidemiology, Modelling and Evaluation, Institute for Global Health; d UCL Respiratory, Division of Medicine, University College London; e Respiratory Medicine, Royal Free Hospital, Royal Free London NHS Foundation Trust; f National Institute for Health and Care Research (NIHR) Health Protection Research Unit (HPRU) in Blood Borne and Sexually Transmitted Infections; g Centre for Clinical Research in Infection and Sexual Health, Institute for Global Health, University College London, London, UK.

**Keywords:** Acute Physiology and Chronic Health Evaluation II, HIV, intensive care, mortality, outcome, survival

## Abstract

**Objective:**

Limited data suggest intensive care unit (ICU) outcomes have improved in people with HIV (PWH). We describe trends in in-ICU/in-hospital mortality among PWH following admission to ICU in a single UK-based HIV referral centre, from 1 January 2000 to 31 December 2019.

**Methods:**

Modelling of associations between ICU admission and calendar year of admission was done using logistic regression with adjustment for age, sex, Acute Physiology and Chronic Health Evaluation II (APACHE II) score, CD4^+^ T-cell count and diagnosis of HIV at/within the past 3 months.

**Results:**

Among 221 PWH (71% male, median [interquartile range (IQR)] age 45 years [38–53]) admitted to ICU, median [IQR] APACHE II score and CD4^+^ T-cell count were 19 [14–25] and 122 cells/μl [30–297], respectively; HIV-1 viral load was ≤50 copies/ml in 46%. The most common ICU admission diagnosis was lower respiratory tract infection (30%). In-ICU and in-hospital, mortality were 29 and 38.5%, respectively. The odds of in-ICU mortality decreased over the 20-year period by 11% per year [odds ratio (OR): 0.89 (95% confidence interval (CI): 0.84–0.94)] with in-hospital mortality decreasing by 14% per year [0.86 (0.82–0.91)]. After adjusting for patient demographics and clinical factors, both estimates were attenuated, however, the odds of in-hospital mortality continued to decline over time [in-ICU mortality: adjusted OR: 0.97 (0.90–1.05); in-hospital mortality: 0.90 (0.84–0.97)].

**Conclusion:**

Short-term mortality of critically ill PWH admitted to ICU has continued to decline in the ART era. This may result from changing indications for ICU admission, advances in critical care and improvements in HIV-related immune status.

## Introduction

The prognosis for people with HIV (PWH) has improved markedly since the introduction of effective combination antiretroviral therapy (ART) in the 1990s, although contemporary data describing this positive trend over time are limited [1–8]. Furthermore, PWH remain susceptible to critical illness related to opportunistic infections due to advanced immunosuppression, bacterial sepsis and tuberculosis at all stages of HIV infection [9–11]. PWH virally suppressed on ART may also experience significant complications of co-morbidities associated with aging and other co-morbid conditions such as chronic liver disease. Thus, PWH continue to require intensive care unit (ICU) admission and represent a not-infrequent clinical encounter for intensivists.

The pattern of critical illness has evolved in the ART era, with a decreasing proportion of AIDS-defining illnesses and an increase in exacerbations of chronic medical conditions, malignancy-related illnesses, surgical complications and adverse effects of ART. Promisingly, studies have reported improvements in short-term mortality (in-ICU mortality and in-hospital mortality) in PWH admitted to ICU, although there continues to be limited data in general on mortality outcomes and related factors. Factors measured at ICU admission associated with increased short-term mortality in PWH include older age, a higher Acute Physiology and Chronic Health Evaluation II (APACHE II) score, a CD4^+^ T-cell count <200 cells/μl, newly diagnosed HIV-1 infection, lack of receipt of ART, respiratory failure, renal failure and the need for mechanical ventilation [1–8,12–23]. The observed improvements in short-term mortality over time are likely due to the evolving indications for ICU admission, and advances in medical management of both HIV-1 infection and critical illness [24–27].

The Royal Free Hospital (RFH) is a large central London teaching hospital with an HIV referral centre that manages one of the largest cohorts of adults living with HIV in the UK (approximately 4000 PWH, June 2020). The RFH ICU is a combined medical and surgical 34-bed unit, which manages approximately 2000 admissions per year. Our main objective was to describe changes in short-term (in-ICU and in-hospital) mortality of PWH admitted to RFH ICU over time. Additionally, we sought to explore the associations between patient factors (age, sex) and clinical factors (APACHE II score, CD4^+^ T-cell count, HIV stage, ART status, advanced HIV, respiratory failure and renal failure) measured at or near the time of ICU admission and these short-term mortality outcomes. To our knowledge, this is the first contemporary study seeking to describe changes in these variables spanning a period as long as 20 years since the establishment of ART as the standard of care for PWH.

## Methods

We retrospectively reviewed electronic records of consecutive, unselected, adult PWH (≥18 years) admitted to RFH ICU over a 20-year period, between 1 January 2000 and 31 December 2019.

Data collected include patient age (years), sex (male/female), and dates of HIV-1 diagnosis, ICU admission, ICU discharge and/or death. Clinical factors captured at the point of ICU admission included primary diagnosis at ICU admission, documented organ failure requiring ICU level support (ICU admission category) and the APACHE II score. The APACHE II score is a composite measure of physiological derangement calculated in the first 24 h of ICU admission, widely accepted to be accurate in predicting ICU mortality in people with HIV since ART became established as the standard of care [4,28–32]. Clinical factors captured up to 3 months prior to ICU admission included CD4^+^ T-cell count (cells/μl), HIV-1 viral load (RNA copies/ml), ART status, hepatitis B and hepatitis C infection status. Those missing data on hepatitis B and/or hepatitis C infection status were assumed to be uninfected, as it was considered unlikely that a positive result would not be recorded. ‘advanced HIV’ was defined as a CD4^+^ T-cell count <200 cells/μl and/or being admitted to ICU with an AIDS-defining illness. HIV stage was classified as ‘newly diagnosed’ if HIV-1 infection was diagnosed at/within 3 months prior to the date of ICU admission, otherwise as ‘established diagnosis’; if only year of HIV-1 diagnosis was available, 1 July of the HIV-1 diagnosis year was assumed. In addition to classifying patients as receiving ART if an ART start date was available, we assumed that patients with a HIV-1 viral load <1000 copies/ml without an ART start date were receiving ART. We sought to capture ethnicity data, but this was largely unavailable as it was not routinely recorded for a majority of the study period.

The RFH's *Freenet ICU* application was used to obtain data on ICU admission details including primary diagnosis, and the departmental *Intensive Care National Audit & Research Centre* (ICNARC) submission records were used to obtain patient demographic data (age, sex), APACHE II score and any documented organ failure requiring ICU-level support. The RFH *WinPath* (CliniSys, Chertsey, UK) blood results system, along with ICU discharge summary documentation available on *Freenet ICU*, and a protected database of all HIV patients managed by RFH, were used to obtain data on primary diagnosis requiring ICU admission, date of HIV-1 infection diagnosis, HIV-1 viral load (RNA copies/mL), CD4^+^ T-cell count (cells/μl), date of initial receipt of ART and hepatitis B and hepatitis C infection status.

We considered two short-term outcomes following ICU admission: in-ICU mortality (defined as a death occurring after initial admission and prior to discharge to a regular hospital bed or from hospital); and in-hospital mortality (defined as any death after initial admission and prior to discharge from hospital, regardless of whether the death occurred in-ICU or after discharge to a regular bed.

### Ethics

This project was registered as a Clinical Audit with RFH in July 2020. It was confirmed to be an Audit by RFH Research and Innovation in October 2021. All data collected were anonymized at the point of capture.

### Statistical analysis

We described characteristics at admission of PWH admitted to ICU between 2000 and 2019, considering only a patient's index admission to ICU. Re-admissions of PWH to ICU in the same and/or subsequent hospital stay(s) were excluded. PWH data were summarized by admission year and categorized into 4-year groups to explore trends in patient characteristics and identify potential nonlinear trends prior to statistical modelling. Year was categorized into 4-year groups to avoid small cell sizes in the exploratory descriptive analyses, while minimizing the loss of information. We conducted statistical tests for trend using the Cochran-Armitage test for categorical variables and the Jonckheere–Terpstra test for continuous variables.

We conducted analyses for short-term mortality outcomes, namely, in-ICU and in-hospital mortality separately using logistic regression, considering only a patient's index admission. It is important to note that those that died in ICU were also considered to have died in hospital, and therefore, are accounted for in both outcomes. Calendar year of a patient's index admission included as a continuous variable was the primary effect of interest to explore trends in the short-term mortality outcomes over the study period. Calendar year was included in models as a continuous variable to avoid model overfitting issues and sparse data bias. Univariable models were used to explore the crude association between short-term mortality outcomes and calendar year as well as several patient characteristics and clinical factors at admission (i.e. age, sex, APACHE II, CD4^+^ T-cell count, HIV stage, whether a patient was receiving ART, respiratory failure and renal failure), all of which are well established as known risk factors for short-term mortality since the establishment of ART as the standard of care in PWH [1–8,12–23]. We also suspected changes in these variables over time, thus were included in univariable analyses. Multivariable models were used to further explore trends in short-term mortality outcomes, adjusting for patient characteristics to assess if changes in the latter explained the trends seen. We first adjusted the calendar year model for patient characteristics identified *a priori* as being likely to be associated with mortality (age, sex) and those that were found to be significantly associated with outcomes in univariable analyses. We then further adjusted for ‘Newly diagnosed’ HIV-1 infection to account for patients with a relatively recent HIV-1 diagnosis who may have a different prognosis than those who have already been living with HIV for >3 months prior to ICU admission. Note that an individual's HIV-1 RNA level was not included in regression analyses due to collinearity with other factors considered (e.g. CD4^+^ T-cell count).

All analyses were conducted using listwise deletion, only including patients with data available on all variables included in analyses. Analyses were performed using R version 4.1.0 (R Foundation for Statistical Computing, Vienna, Austria) with two-sided *P*-values <0.05 considered to be statistically significant.

## Results

### Patient characteristics

During the 20-year study period there were 221 PWH admitted to RFH ICU on 273 occasions; 39 PWH were admitted on more than one occasion. There was strong evidence of an increasing linear trend in the median [interquartile range (IQR)] age at admission over the study period, increasing from 40 years [35–46] in 2000–2003 to 49 years [40–57] in 2016–2019 (*P*-trend = 0.001). The majority of patients admitted were male, ranging from 62.2% in 2008–2011 to 80.0% in 2016–2019; and there was no evidence of a linear trend in the percentage male over the study period (*P*-trend = 0.29). Respiratory failure accounted for the largest proportion of patients among all admissions categories, although the proportion of patients with this markedly decreased between 2000–2003 (90.9%) and 2004–2007 (45.2%); this remained relatively stable after that (global *P*-trend = 0.004 for ICU admission category). Among main diagnosis categories, lower respiratory tract infection (LRTI) was the most common, but patients admitted with this followed a similar pattern between 2000–2003 (50.0%) and 2004–2007 (37.2%) with subsequent stability (global *P*-trend = 0.02 for primary diagnosis category) (Table 1).

**Table 1:** Characteristics of 221 people with HIV admitted to ICU between 2000 and 2019 (index admission only).

*n* (%) or median (IQR)	Overall *n* = 221	2000– 2003 *n* = 24	2004–2007 *n* = 43	2008–2011 *n* = 45	2012–2015 *n* = 44	2016–2019 *n* = 65	*P*-value for trend
Age (years)	45 (38, 53)	40 (35, 46)	44 (38, 49)	44 (41, 51)	46 (38, 53)	49 (40, 57)	0.001
Male	158 (71.5%)	17 (70.8%)	31 (72.1%)	28 (62.2%)	30 (68.2%)	52 (80.0%)	0.29
ICU admission category							
Cardiovascular failure	9 (4.1%)	0 (0%)	0 (0%)	2 (4.5%)	3 (6.8%)	4 (6.2%)	0.004
Multiorgan failure	47 (21.7%)	0 (0%)	9 (21.4%)	17 (38.6%)	8 (18.2%)	13 (20.0%)	
Neurological failure	4 (1.8%)	2 (9.1%)	1 (2.4%)	0 (0%)	0 (0%)	1 (1.5%)	
Observation^a^	38 (17.5%)	0 (0%)	7 (16.7%)	6 (13.6%)	10 (22.7%)	15 (23.1%)	
Renal failure	23 (10.6%)	0 (0%)	6 (14.3%)	4 (9.1%)	5 (11.4%)	8 (12.3%)	
Respiratory failure	96 (44.2%)	20 (90.9%)	19 (45.2%)	15 (34.1%)	18 (40.9%)	24 (36.9%)	
Missing	4	2	1	1	0	0	
Primary diagnosis category							
Cardiovascular	7 (3.2%)	0 (0%)	1 (2.3%)	2 (4.4%)	1 (2.3%)	3 (4.6%)	0.02
Gastrointestinal	24 (10.9%)	0 (0%)	1 (2.3%)	4 (8.9%)	8 (18.2%)	11 (16.9%)	
Infection	30 (13.6%)	7 (29.2%)	3 (7.0%)	8 (17.8%)	5 (11.4%)	7 (10.8%)	
LRTI	67 (30.3%)	12 (50.0%)	16 (37.2%)	10 (22.2%)	10 (22.7%)	19 (29.2%)	
Neurological	23 (10.4%)	2 (8.3%)	5 (11.6%)	6 (13.3%)	4 (9.1%)	6 (9.2%)	
Haemato-oncological^b^	19 (8.6%)	2 (8.3%)	4 (9.3%)	6 (13.3%)	3 (6.8%)	4 (6.2%)	
Renal	13 (5.9%)	0 (0%)	6 (14.0%)	3 (6.7%)	1 (2.3%)	3 (4.6%)	
Other	38 (17.2%)	1 (4.2%)	7 (16.3%)	6 (13.3%)	12 (27.3%)	12 (18.5%)	

LRTI, lower respiratory tract infection.

a Observation: includes planned ICU-level postoperative monitoring of patients as well as ICU-level monitoring of the acutely unwell patient.

b Haemato-oncological includes 14 AIDS-defining cancers (11 non-Hodgkin lymphoma, three Kaposi sarcoma), two non-AIDS-defining cancers (1 Hodgkin lymphoma, 1 metastatic neuroendocrine tumour), and three multicentric Castleman disease.

### Clinical and laboratory characteristics

There was evidence of an increasing linear trend in the median CD4^+^ T-cell count, where the higher median CD4^+^ T-cell counts were observed in the later year groups after 2004–2007, with the highest value occurring in 2012–2015: 212 cells/μl [IQR 46–528] (*P*-trend = 0.01). There was also evidence of an increasing linear trend in the percentage of patients that had an undetectable viral load (≤50 copies/ml), with higher percentages observed in the later year groups, increasing from 16.7% in 2000–2003 to 52.5% in 2016–2019 (*P*-trend = 0.02). In contrast, there was strong evidence of a decreasing linear trend in the median APACHE II score, with lower scores in the later year groups, decreasing from 29 [IQR 23–31] in 2000–2003 to 15 [IQR 10–20] in 2016–2019 (*P*-trend < 0.001) (Table 2).

**Table 2:** Clinical and laboratory characteristics at admission, and length of stay of 221 people with HIV admitted to ICU between 2000 and 2019 (index admission only).

*n* (%) or median (IQR)	Overall *n* = 221	2000–2003 *n* = 24	2004–2007 *n* = 43	2008–2011 *n* = 45	2012–2015 *n* = 44	2016–2019 *n* = 65	*P*-value for trend
APACHE II	19 (14, 25)	29 (23, 31)	25 (21, 29)	17 (12, 23)	18 (14, 22)	15 (10, 20)	<0.001
Missing	13	4	5	3	0	1	
LOS (days)	5 (2, 12)	4 (2, 8)	5 (2, 12)	4 (2, 11)	10 (4, 15)	4 (2, 10)	0.36
CD4^+^ T-cell count	122 (30, 297)	98 (12, 150)	52 (14, 216)	169 (68, 329)	212 (46, 528)	128 (27, 301)	0.01
Missing	13	3	1	1	0	8	
CD4^+^ T-cell count <200 cells/μl	129 (62.0%)	18 (85.7%)	30 (71.4%)	24 (54.5%)	22 (50.0%)	35 (61.4%)	0.03
Missing	13	3	1	1	0	8	
HIV-1 viral load ≤50 copies/ml	94 (45.9%)	3 (16.7%)	13 (34.2%)	26 (59.1%)	20 (45.5%)	32 (52.5%)	0.02
Missing	16	6	5	1	0	4	
Time since HIV-1 diagnosis (months)	65 (2, 163)	57 (6, 92)	28 (1, 97)	86 (28, 128)	116 (3, 207)	63 (0, 216)	0.06
Missing	20	5	1	5	2	7	
Advanced HIV	143 (67.8%)	20 (95.2%)	33 (76.7%)	29 (65.9%)	22 (50.0%)	39 (66.1%)	0.006
Missing	10	3	0	1	0	6	
HIV stage, newly diagnosed	54 (25.1%)	5 (26.3%)	15 (35.7%)	5 (11.1%)	11 (25.0%)	18 (27.7%)	0.85
Missing	6	5	1	0	0	0	
Receiving cART	150 (71.8%)	12 (63.2%)	26 (65.0%)	37 (84.1%)	33 (75.0%)	42 (67.7%)	0.78
Patients with available cART start date	177	16	37	38	37	49	
Missing HIV-1 viral load and cART start date	12	5	3	1	0	3	
Time on cART prior to ICU admission for those with an available cART start date (months)	74 (21, 168)	48 (5, 79)	30 (7, 70)	81 (20, 120)	116 (34, 199)	152 (61, 223)	<0.001
Missing	38	0	4	10	10	14	
HBV positive^a^	20 (9.0%)	0 (0%)	7 (16.3%)	1 (2.2%)	4 (9.1%)	8 (12.3%)	0.35
Patients with data available on HBV status	167	14	35	31	35	52	
HCV positive	31 (14.0%)	2 (8.3%)	5 (11.6%)	8 (17.8%)	9 (20.5%)	7 (10.8%)	0.74
Patients with data available on HCV status	174	14	35	32	37	56	

cart, combination antiretroviral therapy; HBV, hepatitis B virus; HCV, hepatitis C virus; LOS, length of ICU stay.

a HBV positive: hepatitis B virus surface antigen-positive or HBV DNA detectable in serum at time of admission.

The percentage of those ‘newly diagnosed’ with HIV (i.e. diagnosed with HIV-1 infection within 3 months of ICU admission) was quite variable over the study period, with no evidence of a linear trend, where the highest proportion was observed in 2004–2007 (35.7%) and lowest in 2008–2011 (11.1%) (*P*-trend = 0.85). Median time since HIV diagnosis was also variable over the study period, but tended to increase in the latter years, with the shortest median time interval since HIV diagnosis observed in 2004–2007 (28 months [IQR 1–97]) and longest in 2012–2015 (116 months [IQR 3–207]); and there was little evidence of an increasing linear trend (*P* = 0.06). There was strong evidence of a notable decreasing linear trend in the percentage of patients with advanced HIV between 2000–2003 (95.2%) and 2004–2007 (76.7%), and although variable, this percentage tended to be lower more recently (*P*-trend = 0.006). One hundred and fifty (71.8%) patients had an ART start date and/or were assumed to be on ART, and this was relatively stable over the study period with no evidence of a linear trend (*P*-trend = 0.78). Median duration of ART receipt among patients with an ART start date was 74 months [IQR 21–168] overall, and there was strong evidence of an increasing linear trend in the median time interval over the study period with the longest median time interval observed in 2016–2019 at 152 months [IQR 61–223] (*P*-trend < 0.001) (Table 2).

### Short-term mortality outcomes

**Fig. 1:**
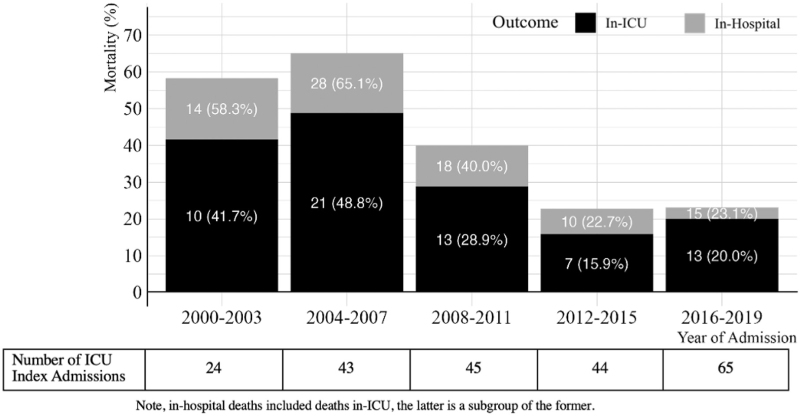
In-ICU and in-hospital mortality by 4-year category.

Overall, in-ICU mortality and in-hospital mortality was 29% (64) and 38.5% (85), respectively, with both outcomes declining over the study period (Fig. 1, note that the count (percentage) of in-hospital deaths included deaths in-ICU, as the latter is a subgroup of the former). The median [IQR] time to death from ICU admission was 4 [1–12] days and 7 [2–19] days for those that died in-ICU and in-hospital, respectively.

### Factors associated with outcomes from ICU admission

In univariable analyses, factors associated with in-ICU mortality were APACHE II score [crude odds ratio (cOR): 1.15 (95% confidence interval (CI): 1.10–1.21); per unit increase] and CD4^+^ T-cell count (cOR: 0.82 (95% CI: 0.72–0.94); log_2_ transformed, per doubling). Patient's age, sex, HIV stage, whether they were receiving ART, respiratory failure and renal failure were not associated with in-ICU mortality. The odds of in-ICU mortality significantly decreased over the 20-year period, with an estimated decrease of 11% per year (cOR: 0.89 (95% CI: 0.84–0.94)). After adjusting for patient demographics and clinical factors, the estimated decreasing trend in in-ICU mortality was attenuated to 3% per year [adjusted OR (aOR): 0.97 (95% CI: 0.90–1.05)] and was no longer statistically significant (Table 3).

**Table 3:** Factors associated with in-ICU and in-hospital mortality using univariable and multivariable logistic regression, odds ratio (OR) [95% confidence interval (CI)].

	Univariable		Multivariable 1^∗^		Multivariable 2^†^	
	OR (95% CI)	*P*-value	OR (95% CI)	*P*-value	OR (95% CI)	*P*-value
In-ICU mortality						
Year (per later year)	0.89 (0.84, 0.94)	<0.001	0.96 (0.89, 1.04)	0.36	0.97 (0.90, 1.05)	0.49
Age (per 5 years)	1.01 (0.88, 1.15)	0.93	1.09 (0.91, 1.31)	0.34	1.08 (0.90, 1.30)	0.48
Sex						
Female	REF		REF		REF	
Male	0.92 (0.49, 1.77)	0.80	1.34 (0.60, 3.12)	0.49	1.31 (0.58, 3.06)	0.52
APACHE II (per unit increase)	1.15 (1.10, 1.21)	<0.001	1.14 (1.08, 1.22)	<0.001	1.14 (1.08, 1.22)	<0.001
HIV stage						
Established diagnosis	REF		–		REF	
Newly diagnosed^§^	1.30 (0.65, 2.53)	0.44			0.96 (0.40, 2.25)	0.93
CD4^+^ T-cell count at admission (cells/μl) (log_2_ transformed)	0.82 (0.72, 0.94)	0.004	0.87 (0.73, 1.02)	0.09	0.86 (0.72, 1.03)	0.09
Receiving cART^¶^						
No	REF		–		–	
Yes	1.38 (0.69, 2.89)	0.38				
Respiratory failure						
No	REF		–		–	
Yes	0.93 (0.51, 1.67)	0.81				
Renal failure						
No	REF		–		–	
Yes	0.85 (0.30, 2.17)	0.75				
In-hospital mortality						
Year (per later year)	0.86 (0.82, 0.91)	<0.001	0.90 (0.84, 0.97)	0.008	0.90 (0.84, 0.97)	0.009
Age (per 5 years)	0.96 (0.85, 1.09)	0.55	1.06 (0.89, 1.26)	0.52	1.06 (0.89, 1.27)	0.51
Sex						
Female	REF		REF		REF	
Male	0.85 (0.47, 1.55)	0.59	1.38 (0.64, 3.04)	0.42	1.41 (0.66, 3.14)	0.38
APACHE II (per unit increase)	1.15 (1.10, 1.21)	<0.001	1.11 (1.06, 1.18)	<0.001	1.11 (1.06, 1.18)	<0.001
HIV stage						
Established diagnosis	REF		–		REF	
Newly diagnosed^§^	1.66 (0.89, 3.11)	0.11			1.55 (0.68, 3.52)	0.30
CD4^+^ T-cell count at admission (cells/μl) (log_2_ transformed)	0.83 (0.73, 0.94)	0.003	0.87 (0.74, 1.03)	0.10	0.90 (0.76, 1.06)	0.20
Receiving cART^¶^						
No	REF		–		–	
Yes	0.92 (0.49, 1.73)	0.79				
Respiratory failure						
No	REF		–		–	
Yes	1.09 (0.83, 1.88)	0.76				
Renal failure						
No	REF		–		–	
Yes	0.67 (0.25, 1.64)	0.41				

∗ Multivariable 1: adjusted factors were year, age, sex, APACHE II, CD4^+^ T-cell count.

† Multivariable 2: adjusted factors were year, age, sex, APACHE II, CD4^+^ T-cell count, HIV stage.

§ Newly-diagnosed if HIV diagnosed within 3 months of ICU admission.

¶ Patients missing cART date with a VL <1000 copies/ml were coded as being on cART.

Similar results were observed for in-hospital mortality. In univariable analyses, the factors predicting in-hospital mortality were APACHE II [cOR: 1.15 (95% CI: 1.10–1.21); per unit increase] and CD4^+^ T-cell count [cOR: 0.83 (95% CI: 0.73–0.94); log_2_ transformed, per doubling]. Patient's age, sex, HIV stage, whether they were receiving ART, respiratory failure and renal failure were not associated with in-hospital mortality. The odds of in-hospital mortality significantly decreased over the 20-year period, with an estimated decrease of 14% per year [cOR: 0.86 (95% CI: 0.82–0.91)]. After adjusting for patient demographics and clinical factors, the estimated decreasing trend was slightly attenuated to 10% per year [aOR: 0.90 (95% CI: 0.84–0.97)] and remained statistically significant (Table 3).

## Discussion

To our knowledge, this is one of very few contemporary, large retrospective studies in a well resourced setting to span a 20-year period of ICU patient care for PWH in the ART era. Our analyses indicate strong evidence of sustained decreases in both in-ICU and in-hospital mortality over the 20-year study period of 11 and 14% per year, respectively, in univariable analyses. After adjustment for patient demographics and clinical factors, the estimated reduction in in-hospital mortality diminished slightly to 10% per year and remained statistically significant. However, the estimated decrease in in-ICU mortality was attenuated and no longer statistically significant, suggesting that the trends seen largely reflect the positive impact on mortality of changes in some of these demographic and clinical factors. The improvement in short-term mortality observed in our study is consistent with trends observed in other studies conducted in the ART era [1–8].

The overall decrease in median APACHE II score over time suggests a reduction in severity of illness at the point of ICU admission. The primary medical diagnosis for ICU admission was LRTI (30.3%), which was mainly due to *Pneumocystis jirovecii* pneumonia (PCP), bacterial pneumonia and pulmonary tuberculosis, but these events represented a decreasing proportion of the overall ICU caseload, where the proportion of LRTI accounted for by PCP also fell markedly over time. A similar trend was also observed for respiratory failure, which was the most frequently documented organ failure requiring ICU-level support. Changes in both these parameters reflect the progressive increase in diversity of the overall caseload of PWH being admitted to ICU, where AIDS-defining illnesses decreased in proportion over time, while the frequency of exacerbations of co-morbidities, malignancy-related complications, surgical admissions and diagnoses unrelated to HIV, increased. Whilst historically, the association of respiratory failure with in-ICU and in-hospital mortality in PWH has been well established, this finding is less prevalent in more recent studies [8–11,18–20] There have been important advances in critical care during the period of this study, which may have had a positive impact on short-term mortality of PWH in ICU. Lung-protective low tidal volume ventilation has been shown to reduce the incidence of acute lung injury in PWH admitted to ICU, which will have influenced the management of those individuals with LRTI, in particular PCP [1,9,13,26,33]. The introduction of this ventilation strategy as the standard of care for critical respiratory illness occurred prior to the study period, and may help to explain why there was no significant association found between short-term mortality and either LRTI or respiratory failure.

The recognition and management of sepsis has also improved markedly over the study period, since the inception of the Surviving Sepsis campaign in 2002 to the latest iteration of sepsis management guidelines, which emphasize the importance of prompt delivery of effective antimicrobial therapy, intravenous fluid resuscitation, monitoring of vital signs and early escalation of patients in septic shock to higher level care where needed [25,27]. This is especially important for PWH in general, where the rates of overwhelming sepsis and bacterial pneumonia are higher at any level of HIV-related immunosuppression when compared to the general population. In addition to earlier escalation of unwell patients by clinicians, a pattern of lower ICU admission APACHE II scores suggest a lowering of the physiological threshold for admission of PWH amongst intensivists, reflecting a shift away the prognostic pessimism of the pre-ART era [6,11,34–37]. The resulting improvement in patient selection for admission to ICU as well as the clearer identification and management of reversible pathology in HIV, may serve to partially explain why the estimated reduction in in-ICU mortality in multivariable analyses was attenuated, but the estimated reduction in in-hospital mortality was preserved.

The trend of both reducing severity of acute illness and state of immunosuppression can also be explained in part by advances in the clinical management of HIV-1 infection. Our findings demonstrate an increase in the number of patients admitted to the ICU who were virally suppressed on ART, and a reduction in HIV-related immunosuppression over time, consistent with previous studies. This was reflected by a greater proportion of patients with an undetectable viral load in later years, as well as a higher median CD4^+^ T-cell count, and thus, a lower proportion of patients with CD4^+^ T-cell count <200 cells/μl. The proportion of PWH receiving ART has markedly increased, influenced by a drive to achieve the UNAIDS 90–90–90 target as well as the paradigm shift brought about by the recommendation of universal treatment for all PWH in 2014 [38,39]. The efficacy and tolerability of ART regimens has also increased over time with the introduction of integrase inhibitors in 2007, which now form the cornerstone of first-line treatment of HIV infection to achieve controlled viral replication [39]. Despite these clear recommendations, there remains uncertainty around when to start ART in newly diagnosed PWH in the ICU. This is important given the legitimate concerns surrounding initiation of ART in critically unwell patients which include adverse effects, drug-drug interactions, drug absorption, induction of viral resistance and the risk of immune reconstitution inflammatory syndrome. There is some evidence to suggest a benefit to commencing ART during the acute critical illness, however we were unable to measure the timing of ART initiation for patients newly diagnosed with HIV-1 infection [17,19].

Late HIV diagnosis, defined as CD4^+^ T-cell count <350 cells/μl or having an AIDS-defining illness regardless of CD4^+^ T-cell count at the time of HIV diagnosis, has been shown to be associated with increased short-term mortality due to a higher incidence of advanced HIV-related events, and is an acknowledged risk factor for in-ICU mortality [40–46]. We have previously demonstrated those PWH with a recent and late diagnosis in this same ICU cohort have a 75% higher risk of in-ICU mortality relative to those who did not have a recent and late HIV diagnosis, where 17 (9/55) per cent of in-ICU deaths were deemed to be preventable [8]. This has important implications for public health efforts aimed at making timely HIV diagnoses to avoid preventable morbidity, mortality and onward transmission of HIV.

We acknowledge several limitations to our study. First, this was a single-centre, retrospective study in an HIV-referral centre in central London, which limits our ability to generalize our findings. Second, the retrospective nature of our study meant that we encountered incomplete data collection from patient records from the early part of the study. We were unable to capture the ethnicity and the risk factor for HIV acquisition for study participants. Unfortunately these data were not routinely captured for the majority of the study period by National Health Service hospitals including our own, though UK data suggest this is improving over time [47]. Third, we were unable to identify a general ICU matched cohort for comparison for this initial analysis.

Future research regarding ICU outcomes for PWH who are admitted to ICU should include prospective validation of the APACHE II, III and IV scores in the era of ART. Further, while short-term outcomes from ICU admission are well described, there is a paucity of literature on longer-term outcomes; for example, at one year. Finally, studies should directly compare short-term and long-term outcomes in PWH with outcomes in the HIV-uninfected general population.

In conclusion, we describe improving short-term outcomes including in-ICU and in-hospital mortality, in PWH admitted to ICU over a 20-year study period. This may be due to factors including advances in critical care, decreasing trends in severity of acute illness and decreasing levels of HIV-related immunosuppression observed over time.

## Acknowledgements

The authors are grateful for the contributions of the Royal Free ICU Data Management Team including Dr Mark de Neef, Dr Nazri Unni and Peggy Tsang. The authors are also grateful for the contributions of the HIV Data Management Team, led by Alan Hunter.

Author contributions: R.F.M., B.A. and C.S. conceptualized the study. T.K., M.S., O.D., S.R. and R.F.M. curated the database. N.B. performed the statistical analyses under the supervision of C.S. T.K., N.B., C.S. and R.F.M. analysed the data, with critical input from O.D., M.S., S.R., A.A., M.C.L., S.B. and B.A. T.K., N.B., C.S. and R.F.M. wrote the first draft of the manuscript. All authors contributed to writing of subsequent drafts and reviewed and approved the final manuscript. T.K. and R.F.M. verified the underlying data and jointly act as guarantors.

Funding disclosure: There was no funding received for this work to disclose

### Conflicts of interest

There are no conflicts of interest.

*Meetings: Limited, preliminary data from this study were presented at the British HIV Association Conference, Manchester, UK (19–21 April 2022): Abstract O07.*
